# Batch production of a silk-elastin-like protein in *E*. *coli* BL21(DE3): key parameters for optimisation

**DOI:** 10.1186/1475-2859-12-21

**Published:** 2013-02-27

**Authors:** Tony Collins, João Azevedo-Silva, André da Costa, Fernando Branca, Raul Machado, Margarida Casal

**Affiliations:** 1 Centre of Molecular and Environmental Biology (CBMA), Department of Biology, University of Minho, Campus de Gualtar, 4710-057, Braga, Portugal

**Keywords:** Biopolymers, Silk-elastin like polymers, pET-*E*. *coli* BL21(DE3), Batch production

## Abstract

**Background:**

Silk-elastin-like proteins (SELPs) combining the physicochemical and biological properties of silk and elastin have a high potential for use in the pharmaceutical, regenerative medicine and materials fields. Their development for use is however restrained by their production levels. Here we describe the batch production optimisation for a novel recently described SELP in the pET-*E*. *coli* BL21(DE3) expression system. Both a comprehensive empirical approach examining all process variables (media, induction time and period, temperature, pH, aeration and agitation) and a detailed characterisation of the bioprocess were carried out in an attempt to maximise production with this system.

**Results:**

This study shows that maximum SELP volumetric production is achieved at 37°C using terrific broth at pH 6–7.5, a shake flask volume to medium volume ratio of 10:1 and an agitation speed of 200 rpm. Maximum induction is attained at the beginning of the stationary phase with 0.5 mM IPTG and an induction period of at least 4 hours. We show that the selection agents ampicillin and carbenicillin are rapidly degraded early in the cultivation and that plasmid stability decreases dramatically on induction. Furthermore, acetate accumulates during the bioprocess to levels which are shown to be inhibitory to the host cells. Using our optimised conditions, 500 mg/L of purified SELP was obtained.

**Conclusions:**

We have identified the optimal conditions for the shake flask production of a novel SELP with the final production levels obtained being the highest reported to date. While this study is focused on SELPs, we believe that it could also be of general interest to any study where the pET (ampicillin selective marker)-*E*. *coli* BL21(DE3) expression system is used. In particular, we show that induction time is critical in this system with, in contrast to that which is generally believed, optimal production being obtained by induction at the beginning of the stationary phase. Furthermore, we believe that we are at or near the maximum productivity for the system used, with rapid degradation of the selective agent by plasmid encoded β-lactamase, plasmid instability on induction and high acetate production levels being the principal limiting factors for further improved production.

## Background

Protein based polymers (PBPs) are a family of artificial biopolymers inspired by Nature. They are adapted from proteins, or more specifically, their amino acid sequences are most typically based on the highly repetitive amino acid sequence blocks of naturally occurring fibrous proteins such as elastin, silk, collagen and/or keratin [1-4]. These repetitive sequences dictate the structure and properties of these materials and are of much interest in the fields of polymer and materials sciences as they offer an exceptional opportunity for the production of polymeric materials in which structure and function are precisely controlled. Indeed, in contrast to conventional synthetic polymers, the composition, sequence and length of PBPs can be strictly controlled, leading to monodispersed, precisely defined polymers which can be biosynthesised in an ecologically friendly manner and which are biodegradable and biocompatible [1,3,5,6]. Furthermore, genetic engineering techniques enable manipulation and modification of the genetically encoded repetitive amino acid blocks and hence of the physical and biological properties of the PBP. In fact, by appropriate amino acid substitutions or modifications, or indeed by use of combinations of natural and/or modified copolymer blocks of distinct properties, one can fine tune the properties and functions of the material being studied [1,3,5,6].

Currently, PBPs are of interest in the fabrication of nano- and micro-structures, with potential for use in tissue engineering, as responsive biomaterials and as functional materials, with, in addition, suggestions being made for their use in biodegradable plastics [3,7-11]. The stimuli (pH, temperature, ionic strength) sensitive capabilities of SELPs enables application in controlled delivery of bioactive agents for intracellular drug and gene delivery, gastrointestinal delivery and tumor targeting. In fact, their potential as controlled release systems for intratumoral gene delivery in cancer gene therapy and in drug delivery has already been shown [6,12]. In tissue engineering, SELP nanofibres have a potential for use as scaffolds in tissue repair and in the fabrication of soft connective tissue substitutes [13,14]. Finally, optically transparent films produced from SELPs have been suggested for use in synthetic corneas, intraocular lens, contact lens and even in ophthalmic drug delivery systems [15].

A series of PBP diblock copolymers, consisting of multiple blocks of the silkworm silk consensus sequence GAGAGS in various combinations with a variant (VPAVG) of the natural mammalian elastin repetitive sequence block VPGVG, have recently been synthesised [16]. This integration of the high tensile strength silk blocks with the highly resilient elastin-like blocks in various combinations allows for the controlled fabrication of a range of novel structures of diverse mechanical and biological properties [3,13,17].

The genes encoding the newly designed SELPs have been synthesised and the novel recombinant polymers expressed in *E*. *coli* by use of the pET25b-*E*. *coli* BL21(DE3) expression system, with yields of approximately 150 mg/L being reported [16]. This T7-based expression system is one of the most widely used for recombinant protein expression yet it often suffers from low yields of protein product [18-20]. Indeed, it is insufficient polymer yield which constitutes one of the major obstacles to further development and commercial viability of PBPs. Thus it is essential that process optimisation, to maximise productivity of the novel SELPs, is embarked upon, with parameters such as cultivation mode, expression host and vector, medium composition, inducer, inducer concentration, elapsed fermentation time (EFT) at induction, induction period and environmental factors such as temperature, pH, oxygenation and agitation rates being important. In fact it is recommended that each and all of these parameters be optimised for each particular expression system, protein product and cultivation mode used.

As a first step in our undertaking to study and optimise the production levels of the novel SELPs, we investigated batch fermentation in shake-flasks with the pET25b – *E*. *coli* BL21(DE3) expression system. Both an in-depth empirical study using the one-factor-at-a-time (OFAT) approach to investigate all important parameters and a non-empirical investigative study were carried out to optimise the productivity of the process as well as to better understand the factors limiting further improved batch production levels of the SELP studied.

## Results and discussion

**Figure 1:**
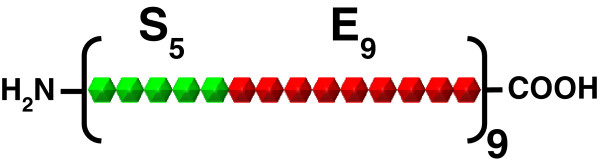
**Schematic representation of the SELP**-**59**-**A construct.** The polymer contains 9 repeats of a monomeric unit which itself consists of 5 repeats of the silk consensus sequence GAGAGS (S_5_, green) linked to 9 repeats of the elastin-like sequence VPAVG (E_9_, red). NH_2_ and COOH correspond to the amino and carboxy termini, respectively.

In this study we attempted to maximise the shake flask volumetric production levels of a novel SELP (SELP-59-A, Figure 1) expressed with the pET-*E*. *coli* BL21(DE3) system. This bacteriophage T7 polymerase/promoter based expression system is one of the most widely used for recombinant protein expression, with variable yields being reported and being dependent on the target protein and the process parameters used. The most commonly employed shake flask production approach uses lysogeny broth (LB) with isopropyl β-D-1-thiogalactopyranoside (IPTG) induction at the mid-exponential phase of growth [18,20-22]. Recently however, richer media have gained favour, with terrific broth (TB) and super broth (SB) as well as variants of these being the most common [20,23,24]. Nevertheless, even with these media, shake flask production levels of proteins and enzymes using these approaches are typically only in the low mg/L range [18-20,23-25]. More specifically, in relation to PBPs, 5 to 300 mg/L have been typically documented for silk-like and elastin-like PBPs [18,19,25] with a 1.6 g/L production even being reported for the latter [26]. On the other hand, production levels of only 20 – 30 mg/L are typically reported with SELPs [27,28] whereas we recently obtained approximately 200 mg/L of SELP-59-A by use of an autoinduction approach [16].

In the present study, we endevoured to: 1) maximise the SELP-59-A production levels and 2) obtain a better understanding of batch production approaches and of the parameters limiting these when using the pET (ampicillin selection marker) – *E*. *coli* BL21(DE3) expression system. All important parameters influencing volumetric production levels in shake flasks were investigated: medium and medium composition, initial pH of the medium, incubation temperature, culture volume to flask volume ratio, agitation rate, IPTG induction concentration, elapsed fermentation time (EFT) at induction and induction period. The effects of varying each of these parameters on the biomass and SELP-59-A production levels were monitored and compared in an attempt to optimise these and to better understand their impact in shake flask productions. Biomass levels were monitored by measuring the dry cell weight (DCW) whereas SELP-59-A volumetric (mg SELP/L production culture) and specific (mg SELP/g biomass) productivities were calculated from a comparative sodium dodecyl sulphate polyacrylamide gel electrophoresis (SDS-PAGE) wherein the SELP-59-A band intensities were compared to that of a standard sample of known concentration. While the most relevant factor for production optimisation is the volumetric productivity, the monitoring of biomass production and SELP-59-A specific productivity allows for a better understanding of the influence of host cell growth and host cell production efficiency on this.

### Culture medium optimisation

**Figure 2:**
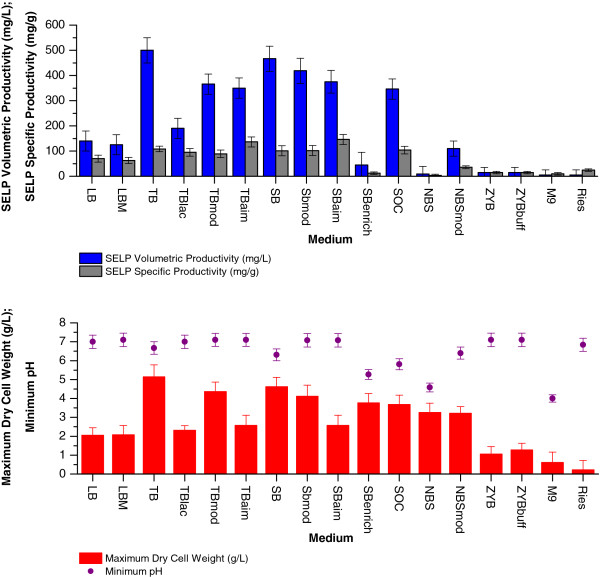
**Media optimisation.** Comparison of maximum SELP-59-A volumetric (blue) and specific (grey) productivities (top), maximum biomass levels (red) and minimum pH (purple) measured (bottom) as a function of the culture medium used. The maximum biomass values represent the highest dry cell weights (DCW, g/L) measured over the course of the experiment. The SELP-59-A volumetric and specific productivities were estimated from comparisons of band intensities on a sodium dodecyl sulphate polyacrylamide gel with a band of known concentration.

As a first step in our optimisation study we investigated the production medium wherein a number of frequently used complex media as well as some defined media were examined. Figure 2 shows the maximally attained biomass and SELP-59-A production levels as well as the lowest pH measured during the cultivations with the various media. It can be seen that both TB and SB are the most suited for SELP-59-A production under the conditions used, with the commonly employed LB and its variant LBM allowing for only approximately 30% of the volumetric productivities of these. Volumetric productivities of approximately 500 mg SELP-59-A per litre of production culture were obtained for both TB and SB, with the variants of these (TBlac, TBmod, TBaim etc.) and all other media tested resulting in reduced amounts (Figure 2). On the other hand, specific productivities were found to be similar among all rich media tested, with approximately 100 mg SELP-59-A per g DCW of biomass being observed for TB, TBlac, TBmod, SB, SBmod and SOC. The auto-induced media tested allowed for slightly increased specific productivities (approx. 120 mg/g) whereas those with a lower nutrient content (i.e. LB, LBM, NBS, M9, Ries) or high carbon source content (SB enrich, NBS, M9) had reduced specific productivities.

The nutrient rich, carbon source supplemented and buffered nature of TB and SB obviously confer these with advantages over the other media tested in terms of both host cell growth and specific SELP-59-A productivity. The high content of yeast extract, tryptone peptone and phosphates enables high biomass dry cell weights and high specific productivities, while the reduced accumulation of overflow metabolites, by virtue of using glycerol as the carbon source supplement, in conjunction with the use of a phosphate buffer, reduces unfavourable pH changes. In fact, the low pHs encountered with SBenrich, SOC, NBS and M9 are most probably resultant of the high concentrations of the overflow metabolite accumulating carbon source used (i.e. glucose) or/and the poor buffering ability of these media. Indeed in those cases where the highest glucose concentrations were used (SBenrich, NBS and M9) both the cell growth and specific productivity were strongly impaired. What is more, the glycerol supplemented and buffered nature of TB and SB also delays the onset of a pH rise; this being in contrast to LB, LBM, TBmod, TBaim, SBmod, SBaim, ZYB and ZYBbuff where a pH increase is observed earlier in the cultivations. In these cases, in the absence of sufficient levels of the added carbon source, scavenging for carbon via peptide and protein degradation probably occurs, leading to subsequent ammonium release and hence also the observed pH rise [29].

The auto-induction media investigated (TBlac, TBaim and SBaim), which allow for automatic induction of SELP production on the formation of allolactose from the lactose present, also led to reduced volumetric production levels under the conditions used as compared to TB and SB (Figure 2). Previous studies have indicated improved production with autoinduction media when using reduced oxygen availability [30] yet our investigation with shake flask volume to culture volume ratios of 10:1 and 4:1 both led to reduced final cellular densities and SELP-59-A volumetric productivities as compared to TB and SB at high oxygenation rates (i.e. 10:1). The reduced biomass dry cell weights observed are suggestive of a premature induction of recombinant protein production, hence placing a considerable metabolic burden on the cells and reducing growth. A fine tuning of the metabolic control of the induction, with in particular an optimisation of the respective concentrations of glucose, glycerol and lactose, may allow for improved yields using this approach [24].

ZYB and the defined media tested did not support satisfactory culture growth under the conditions used and hence SELP yields were low (Figure 2).

In an attempt to further improve the production in TB and SB we investigated these media further, initially investigating the effect of varying the sugar supplements used. Glycerol, glucose and fructose were examined at concentrations from 0 to 20 g/L but no significant increases in biomass or SELP yields were detected under the conditions used. Indeed, at the higher concentrations of these carbon sources, a drop in pH to below pH 6.0 was accompanied by a reduced cellular density and drastically reduced SELP production (> 90% loss). Interestingly, differences in final cell density and recombinant protein production might have been expected with the different carbon sources tested as these have dissimilar uptake rates and lead to different byproducts in *E*.*coli*[23,31]. However, with the complex rich media used in this study, the sugar supplements do not constitute the only source of carbon present [23] and hence these have a reduced effect. In fact, we even found that TB without a sugar supplement led to a final cell density of as much as 75% of that in sugar supplemented media. The effects of varying the concentrations of yeast extract and bacto tryptone in TB were also investigated, but again no increases in SELP production levels were noted. Ammonium and amino acid additives were then examined as previous studies have indicated the positive effects of ammonium as a nitrogen source, even in the presence of excess organic nitrogen [32]. Furthermore, due to the highly repetitive nature of the amino acid sequences of PBPs, the potential benefits of exogenously added amino acids can be easily understood and has been previously shown. Chow et al. [26] documented increased PBP production in rich media supplemented with proline and alanine, while Tuite et al. [33] showed the beneficial effects of methionine and isoleucine supplementation on *E coli* growth in minimal media due to reduced acetate toxicity. In contrast, in our study, addition of ammonium or various amino acids (V, P, A, G, S, M, I), or of supplements such as NaCl or magnesium, did not have any significant effect on production levels under the conditions used. These ingredients are most probably already present in sufficiently high concentrations in the rich media used here. In fact, this inability to further improve SELP production by media composition manipulation, under the conditions used, points to effects other than media composition being limiting to further recombinant protein production.

Finally, in relation to the culture media, we would also like to re-emphasise the importance of careful media preparation, in particular the importance of sterilising the phosphates separate from the remaining ingredients so as to prevent the formation of various poorly soluble phosphate complexes of reduced bioavailability e.g. calcium, magnesium, manganese, and/or iron phosphate precipitates. Indeed we have noted three to four fold improvements in volumetric production levels with appropriately prepared rich media as compared to those wherein all ingredients were autoclaved together.

### Optimisation of environmental parameters

*E*. *coli* is a facultative anaerobe with an optimal growth temperature of 37°C and optimal pH range of between 6.0 and 7.5. It is generally grown under aerobic conditions as anaerobic growth yields less energy for metabolic processes such as protein synthesis [34].

**Figure 3:**
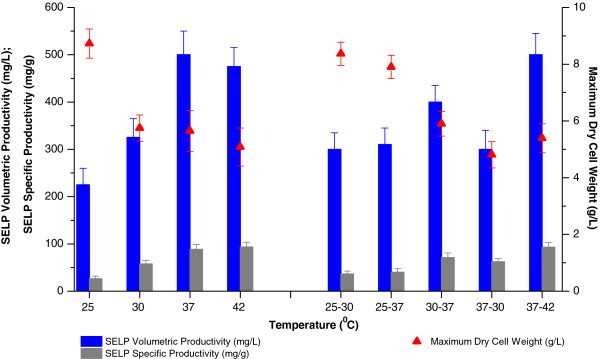
**Effect of temperature on biomass and SELP production.** Comparison of maximum SELP-59-A volumetric (blue) and specific (grey) productivities as well as maximum biomass levels measured (red) as a function of the incubation temperature used. The right hand side of the figure (25–30, 25–37, 30–37, 37–30, 37–42°C) represents the temperature shift experiments: the initial temperatures used and the temperatures after induction. The maximum biomass values represent the highest dry cell weights (DCW, g/L) measured over the course of the experiment. The SELP-59-A volumetric and specific productivities were estimated from comparisons of band intensities on a sodium dodecyl sulphate polyacrylamide gel with a band of known concentration.

An examination of the effects of temperature on SELP-59-A production in TB (Figure 3) indicated, as expected, 37 to 42°C as being the most desirable. Highest final biomass production was observed at 25°C but this was also characterised by a strongly reduced specific productivity with, as a result, the volumetric productivity being only 45% of that at 37°C. Indeed, specific productivity was found to increase with increasing temperature up to 37–42°C, in agreement with a previous suggestion that the T7-based expression system is repressed at low temperatures [35]. Finally, our studies investigating various pre and post induction temperatures showed that while increased post induction temperatures did allow for increased specific productivities, these were insufficient to allow for improvements in volumetric productivities as compared to production at 37°C (Figure 3).

**Figure 4:**
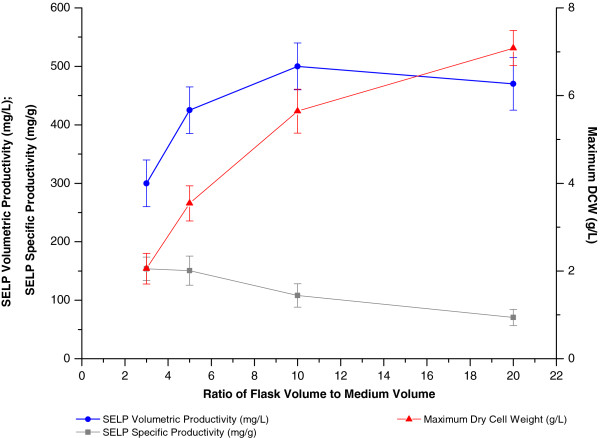
**Effect of the flask volume to culture medium volume ratio on biomass and SELP production.** Comparison of maximum SELP-59-A volumetric (blue) and specific (grey) productivities as well as maximum biomass levels measured (red) as a function of the flask volume to medium volume ratio. The maximum biomass values represent the highest dry cell weights (DCW, g/L) measured over the course of the experiment. The SELP-59-A volumetric and specific productivities were estimated from comparisons of band intensities on a sodium dodecyl sulphate polyacrylamide gel with a band of known concentration.

**Figure 5:**
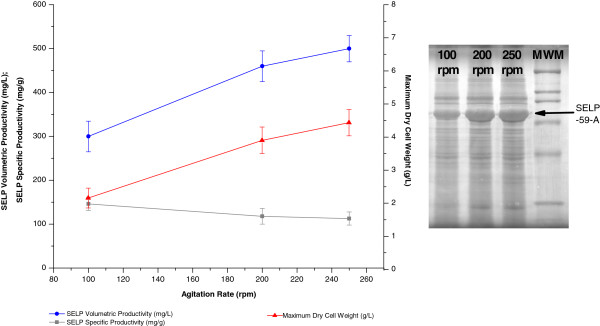
**Effect of agitation rate on biomass and SELP production.** Comparison of maximum SELP-59-A volumetric (blue) and specific (grey) productivities as well as maximum biomass levels measured (red) as a function of the rate of culture agitation. The maximum biomass values represent the highest dry cell weights (DCW, g/L) measured over the course of the experiment. The SELP-59-A volumetric and specific productivities were estimated from comparisons of band intensities on a sodium dodecyl sulphate polyacrylamide gel with a band of known concentration. On the right is shown the SDS-PAGE analysis of the effect of the agitation rate (150, 200 and 250 rpm) on SELP-59-A production. The position of the SELP-59-A band is indicated. A broad range SDS-PAGE molecular weight marker (Biorad) is also shown (MWM). rpm: rotations per minute.

Aeration, or oxygen availability, and mixing efficiency are critical parameters for the growth and metabolism of *E*. *coli*. In shake flask productions, the flask volume to liquid volume ratio and the agitation rate are the modifiable parameters which influence the culture mixing and aeration and hence were optimised in our study (Figures 4 and 5). Increases in the values of these parameters, which lead to an improved aeration and oxygen transfer efficiency, were found to allow increased host cell growth. However, this increase was counterbalanced by a reduced productivity of the cells and resulted in a maximum SELP volumetric production being observed at a flask volume to culture volume ratio of 10:1 and an agitation rate of 200 to 250 rotations per minute (rpm). This saturating effect on SELP productivity at the higher volume:volume ratios and agitation rates tested indicates the presence of other limiting factors under the conditions used, including possibly a greater production of toxic byproducts at the higher growth rates achieved.

Finally, as expected, optimal biomass and recombinant protein production was attained at near neutral pHs, with little variation between pH 6.0 and 7.5. Decreases in production levels were indeed observed outside this range, with a decrease to approximately 60% of the maximum being already observed at initial culture pHs of 5.5 and 8.0.

### Induction optimisation

**Figure 6:**
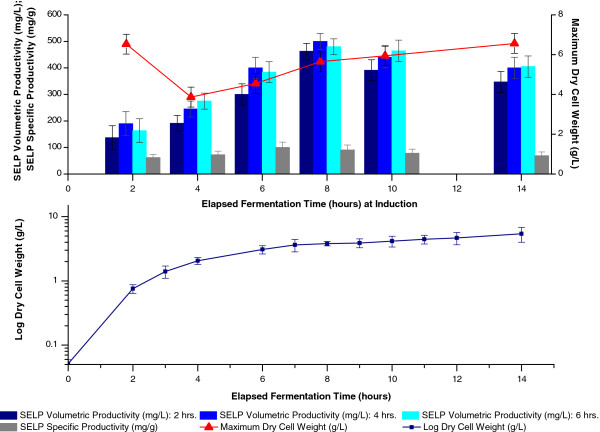
**Effect of elapsed fermentation time at induction and induction period on biomass and SELP production.** Comparison of maximum SELP-59-A volumetric (blue) and specific (grey) productivities as well as maximum biomass levels measured (red) as a function of the elapsed fermentation time (EFT) at induction (2–14 hrs.incubation) and the induction period (2, 4 and 6 hrs.) (top). Growth curve of uninduced *E*.*coli* BL21(DE3) for comparison of the elapsed fermentation time at induction with the stage of growth (bottom). Cultures were induced with 1 mM IPTG. The EFTs at induction correspond to induction during the exponential (2 hrs. EFT, 0.75 g/L DCW), declining exponential (4 and 6 hrs. EFT, 2 and 3 g/L DCW), early stationary (8 hrs. EFT, 4 g/L DCW), stationary (10 hrs. EFT, 4 g/L DCW) and late stationary (14 hrs. EFT, 5 g/L DCW) phases of growth. The maximum biomass values represent the highest dry cell weights (DCW, g/L) measured over the course of the experiment. The SELP-59-A volumetric and specific productivities were estimated from comparisons of band intensities on a sodium dodecyl sulphate polyacrylamide gel with a band of known concentration.

**Figure 7:**
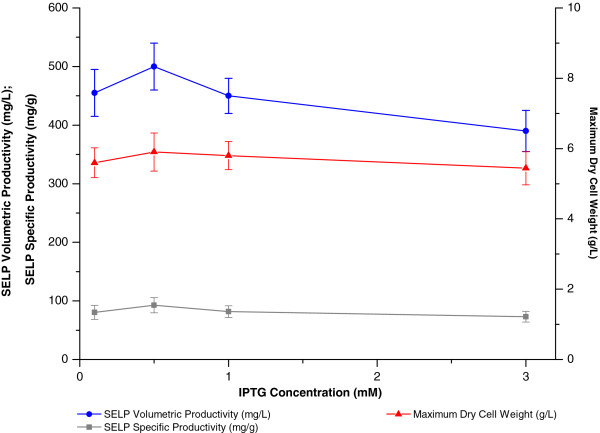
**Effect of inducer concentration on biomass and SELP production.** Comparison of maximum SELP-59-A volumetric (blue) and specific (grey) productivities as well as maximum biomass levels measured (red) as a function of isopropyl β-D-1-thiogalactopyranoside (IPTG) concentration. The maximum biomass values represent the highest dry cell weights (DCW, g/L) measured over the course of the experiment. The SELP-59-A volumetric and specific productivities were estimated from comparisons of band intensities on a sodium dodecyl sulphate polyacrylamide gel with a band of known concentration.

The effects of the growth phase at induction, the induction period and concentration of the inducer were next examined. Most commonly, induction is carried out during the exponential growth phase when cells are most actively dividing and when the protein expression machinery is believed to be the most active [18,21,22,36]. In contrast, our studies indicated that maximum SELP volumetric productivity is achieved by induction at the beginning of the stationary phase (8 hrs. EFT, 4 g/L DCW) when the growth rate had slowed to approximately 0.1 hr.^-1^ (Figure 6). Nevertheless, in common with previous studies, we also found that at least 4 hours induction is required (Figure 6) and that little variation in productivity is observed with IPTG concentrations of between 0.1 and 1 mM IPTG (Figure 7). This is within the range of 0.1 – 3 mM IPTG which is generally employed.

**Figure 8:**
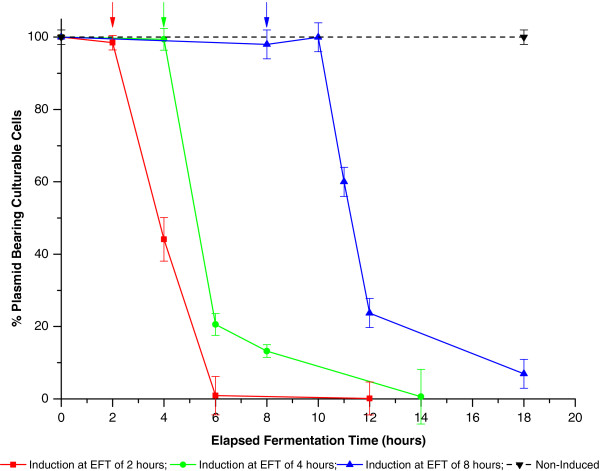
**Plasmid stability**, **without induction and with induction at various elapsed fermentation times (EFTs).** Cultures induced during the exponential (2 hours EFT, biomass DCW of approx. 1 g/L, red), declining exponential (4 hours EFT, biomass DCW of approx. 2 g/L, green) and early stationary (8 hours EFT, biomass DCW of approx. 4 g/L, blue) phases of growth as well as non-induced cultures (black) are compared. The induction points (EFT) are indicated by arrows. The optimised shake flask conditions for SELP production as developed in the present study were used.

Under the conditions used in this study both the biomass and specific productivities, and hence also the volumetric productivity, were found to be maximum when induction was carried out at the beginning of the stationary phase (Figure 6). Indeed, the specific productivity was also found to be high when induced near the end of the declining exponential phase (6 hrs. EFT, 3 g/L DCW) but the higher biomass levels achieved during the stationary phase resulted in a superior final volumetric productivity when induced during this phase. In fact, biomass growth was found to be strongly reduced following induction which is probably resultant of a diversion of cell resources to protein expression, hence preference was given to inducing later in the growth curve when biomass readings were higher. Interestingly, a high final biomass DCW was also observed after induction during the exponential phase of growth (2 hrs. EFT, 0.75 g/L DCW) but rapid plasmid loss on induction, with complete loss of the expression plasmid after 4 hours (Figure 8), indicates this to be due to outgrowth of non-producing cells. It can also be seen that induction during the stationary growth phase allows for an improved plasmid stability (approx. 100% of culturable cells still retain plasmids after 2 hours induction) as compared to induction earlier in the growth curve. Indeed this higher plasmid stability may be responsible for the enhanced specific productivity noted for this condition and a decoupling of biomass build up and recombinant protein production during this phase may be responsible for this effect [37-39]. It is speculated that inherently lower energy requirements during the slowing growth (growth rate of approx. 0.1 hr.^-1^) as opposed to during exponential growth, as well as an improved resistance to stress of cells in the latter phases of the growth curve [38], may enable the cells to better overcome the metabolic burden of the recombinant protein production and hence permit the observed improved production and plasmid stability on induction during the early stationary phase of growth.

**Figure 9:**
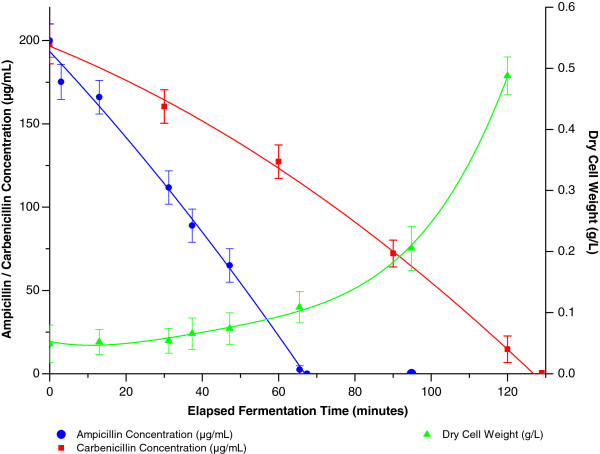
**Ampicillin concentration, carbenicillin concentration and biomass levels as a function of the elapsed fermentation time.** The optimised shake flask conditions for SELP production as developed in the present study were used. Ampicillin concentration is shown in blue, carbenicillin in red and the dry cell weight is shown in green.

Recombinant protein expression places considerable pressure on the host cell and as a result induction can lead to reduced culture growth and rapid plasmid loss (Figures 6 and 8). Typically the selective agent used would be expected to exert a stabilising influence on plasmid maintenance but as can be seen from Figure 9 both ampicillin and the semi-synthetic antibiotic carbenicillin are rapidly degraded during, respectively, the first hour and two hours of cultivation in our studies. β-lactamase, which enacts resistance by degradation of the antibiotics tested, accumulates during cell growth and eventually reaches levels which lead to complete depletion of the antibiotics in the medium [40]. This removal of the selective agent obviously facilitates the observed plasmid loss in culturable cells and would also increase the risk of contamination. Surprisingly, even though we used initial antibiotic concentrations of 200 μg/mL (4-times the recommended concentration) we already observed a complete exhaustion of ampicillin at a biomass DCW of 0.1 g/L and carbenicillin at a biomass DCW of 0.5 g/L (Figure 9), as well as an almost immediate exhaustion in cultures directly inoculated with an overnight preculture solution. This indicates the importance of using β-lactamase free cell re-suspensions for culture inoculation but also allows one to question the real effectiveness of using ampicillin or carbenicillin as selective agents with the expression system and conditions used. In fact we have even observed similar SELP production levels in the presence and absence of these antibiotics.

Finally, in contrast to that observed by Guda et al. [7], where optimum PBP production occurred with uninduced cultures when using the pET11d – *E*. *coli* HMS174(DE3) expression system, we observed little or no production even after 72 hours cultivation in the absence of induction. This may be due to the different expression systems used (*E*. *coli* BL21(DE3) is an *E*. *coli* B strain derivative whereas *E*. *coli* HMS174(DE3) is derived from K-12) or, more possibly, differences in the composition and/or quality of the media ingredients employed. The complex unrefined nature of the media ingredients used (namely yeast extract and tryptone peptone) results in an inherent variation in the composition of these according to the commercial source and production batch and we have previously noted unintended induction for various proteins as a result of these disparities. Indeed others have also noted this and indicated that low variable levels of lactose contamination in yeast extract and to a lesser extent also tryptone peptone may be responsible for this unintended induction in rich media [24,41].

### Process characterisation: optimised conditions

**Figure 10:**
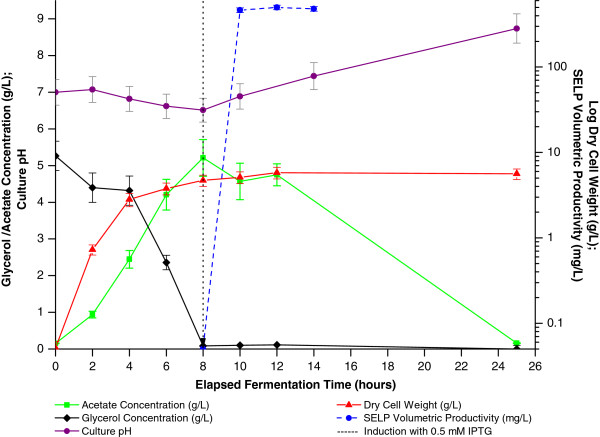
**Characterisation of optimised production process.** Monitoring of glycerol concentration (black), acetate concentration (green), biomass levels (red), SELP volumetric productivity (blue) and pH (purple) as a function of the elapsed fermentation time (EFT) with the optimised process conditions for SELP production. The dotted black vertical line at 8 hours marks the elapsed fermentation time at induction with 0.5 mM IPTG.

**Figure 11:**
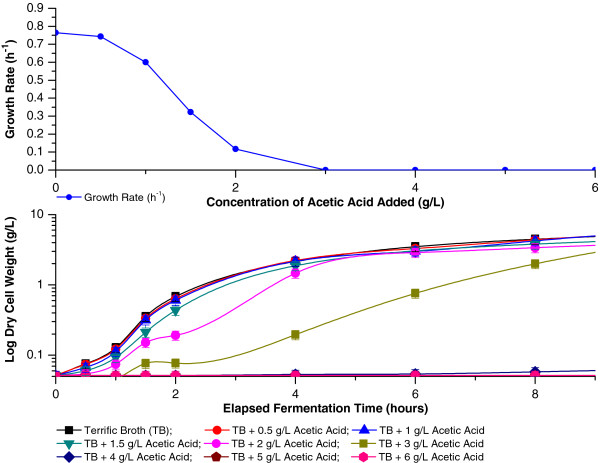
**Effect of acetic acid on growth of *E. coli *BL21 DE3(+)/pET25b/SELP-59-A.** Effect of acetic acid, added at an elapsed fermentation time of 0 hours, on growth of *E*. *coli* BL21 DE3(+)/pET25b/SELP-59-A cultivated under the optimised shake flask conditions of the present study. The variation in the initial growth rates (measured over the initial 30 minutes of incubation) as a function of the amount of acetic acid added (top), and biomass levels (as measured by g/L DCW) over the course of the bioprocess (bottom) are shown.

Characterisation of the optimised bioprocess (i.e. TB, pH 7.0, 37°C, flask vol.:liquid vol. ratio of 10:1, 200 rpm, 0.5 mM IPTG at the beginning of the stationary phase of growth for ≥ 4 hours) identified high acetate levels (Figures 10 and 11) in addition, as discussed above, to plasmid instability (Figure 8) and rapid ampicillin degradation (Figure 9) as being the principal factors limiting biomass and SELP-59-A production levels. From Figure 10 it can be seen that glycerol levels rapidly decrease during the process with a concomitant accumulation of acetate to approximately 5 g/L and a decrease of pH. At low glycerol concentrations the cells then switch to a utilisation of the accumulated acetate with an accompanying pH increase. Interestingly, it can also be seen that our empirical study identified the optimal induction time as that corresponding to the point of glycerol depletion and hence it is the accumulated acetate which provides the carbon source during recombinant protein production. Finally, phosphate (PO^4-^) and nitrogen (NH_3_) levels are not believed to have been limiting during the bioprocess with, respectively, approximately 80% (75 mM) and almost 100% (500 mM) of the initial concentrations of these being detected after 12 hours incubation.

Acetate is produced as an extracellular co-product of aerobic fermentation by *E*. *coli* in the presence of an excess of carbon source when carbon flux into the cells exceeds the capacity of the central metabolic pathways [42]. It is well known to negatively affect *E*. *coli* growth and recombinant protein production [43,44]. However, the effects, and indeed also the levels of acetate produced, depend on the strain used as well as on the medium and growth conditions and importantly also the carbon source [43]. We investigated the effects of various levels of acetic acid, added at the initiation of cultivation, on the growth of our recombinant host under the conditions optimised during the empirical study. From Figure 11 it can be seen that already at 1 g/L acetic acid a slight decrease in the initial growth rate is observed, with much stronger effects being noted at higher concentrations. Indeed, 1 to 4 g/L acetic acid appear to have bacteriostatic effects only, with concentrations of 5 g/L or higher having bactericidal effect under the conditions used (Figure 11, bottom). Interestingly, our studies also indicated a greater acid tolerance by cells in the latter phases of the growth curve, with at least 6 and 7 g/L acetic acid being required for induction of a bactericidal effect when added at 2 and 4 hours culture incubation, respectively (results not shown). This increased resistance may possibly be a result of an induced acid tolerance of cells following exposure to the increasing concentrations of the acid produced during growth [45].

Analysis of shake flask cultures of glucose or fructose supplemented media showed, as expected, a more rapid depletion of these sugars (exhausted after 4 hours incubation) and a correspondingly more rapid acetate accumulation as compared to when glycerol is used (results not shown). More interesting however, is that in the absence of a sugar supplement, acetate was still produced with a maximum of around 3 g/L being accumulated, thereby indicating the presence of an acetogenic carbon source in the TB medium used. The high concentrations of the complex components; yeast extract and peptones, used in this media are probably the sources of this acetogenic carbon source. Hence it can be seen that the use of a complex rich medium for the batch cultivation of *E*. *coli* leads to an uncontrolled growth and a production of inhibitory concentrations of acetate which limits further cell growth and hence also protein production. It can be understood how this can be the major limiting factor for further improving production levels using the shake flask mode of cultivation and how, as observed in this study, the use of additives such as amino acids, ammonia, various carbon sources etc. have little or no effect.

## Conclusions

In this study we have carried out a comprehensive and detailed process characterisation to identify optimal conditions as well as key parameters influencing *E*. *coli* BL21(DE3) growth and SELP product yield in shake flasks. While the OFAT approach used here does not allow for an analysis of interactions between parameters and is less efficient as compared to a rational experimental design approach, it has nonetheless enabled for a better understanding of the effects of each parameter on the production process. The optimum medium, environmental conditions and induction conditions allowing for maximum product yield in shake flask cultivations have been determined and allow for the obtention of 500 mg of purified SELP-59-A per litre of production culture. This is the highest production level reported to date for SELPs, it is almost 20-fold higher than that reported for SELP production by other groups [27,28] and is 2.5-fold higher than that previously reported for SELP-59-A [16]. We have clearly shown that when using the expression system of this study, optimal production is observed using rich, sugar supplemented, buffered media (such as TB or SB) and environmental conditions close to the physiological conditions of the producing organism (i.e. 37°C, near neutral pH). Good mixing and aeration, via high rates of agitation and high flask volume to culture volume ratios, were found to be important for improved production, and induction at the beginning of the stationary phase was shown to be critical for maximising this.

We believe that the production levels of the novel SELP attained in this study using the pET25b-*E*. *coli* BL21(DE3) expression system are close to the limits of that achievable with shake flask cultivations. Plasmid instability on induction, facilitated by a rapid depletion of the selective agent, as well as acetate accumulation have been identified as factors limiting further increases in production levels. The former has a negative effect on the specific productivity whereas the latter limits the biomass levels achieved as well as possibly also the specific productivity. Plasmid instability is probably a result of the metabolic burden placed on cells during recombinant protein production whereas acetate accumulation is a result of the uncontrolled, high growth rates observed with the complex rich medium used. It is difficult to overcome these limitations using the expression system and shake flask cultivation mode used in this study and hence other approaches are required. In an attempt to further improve our production levels we are currently investigating the use of the fed-batch approach for control and reduction of the acetate accumulation as well as the use of more appropriate selection pressures (e.g. antibiotic resistance not based on degradation and/or toxin/antitoxin postsegregational suicide systems) for improving plasmid stability.

Finally, we would like to note that we have made similar observations, to those described here for SELP-59-A, for the optimum production conditions for both a globular protein (a thiol-disulphide oxidoreductase) and another synthetic fibrous protein (a silk-like polymer). Again here, rich, buffered, glycerol supplemented media at neutral pH, with high rates of agitation and a high ratio of flask volume to medium volume, in addition to induction at the beginning of the stationary phase for at least four hours, was found to allow for highest shake flask production levels. Indeed, these observations point to a potential universal applicability of our proposed conditions for protein expression using the pET-*E*. *coli* BL21(DE3) expression system with ampicillin or carbenicillin as the selection agents. We suggest that the conditions described constitute good starting conditions for the shake flask production of any protein using this system.

## Methods

### SELP construct and expression system

The novel SELP construct used for this study was SELP-59-A (Figure 1). This polymer contains 9 repeats of a monomeric unit which itself consists of 5 repeats of the silk consensus sequence GAGAGS linked to 9 repeats of the elastin-like sequence VPAVG. The gene for this SELP had already been synthesised, inserted in the pET25b(+) expression vector (Novagen) and expressed in *E*. *coli* BL21(DE3) [16]. This expression system was used throughout the study for the optimisation of SELP production in shake-flasks as described below.

### Shake-flask production optimisation

The parameters investigated in this study were: medium and medium composition, initial pH of the medium, incubation temperature, culture volume to flask volume ratio, agitation rate, IPTG induction concentration, elapsed fermentation time (EFT) at induction and induction period. The ‘one-factor-at-a-time’ (OFAT) approach was used whereby the parameter being investigated was varied while all other parameters were kept constant at the standard conditions as described below. While being less powerful than a rational experimental design approach it does allow for an individual analysis of the various parameters and a clear observation of the general trends and effects of each.

All productions were run for 30 hours and were repeated at least two times, biomass and SELP-59-A production levels and medium supernatant pH were monitored and compared throughout. The biomass production values were determined from dry cell weight (DCW) measurements (see below for details). Relative SELP-59-A production values were estimated from a comparative sodium dodecyl sulphate polyacrylamide gel electrophoresis (SDS-PAGE, see below for details) wherein the SELP-59-A band intensities for the variant conditions tested for a particular parameter were compared to that of the optimised standard condition. The volumetric productivity (mg SELP/L production medium) and specific productivity (mg SELP/g DCW) were not directly measured but were calculated from the SDS-PAGE comparisons wherein the optimised standard condition represented a volumetric productivity of 500 mg/L and a specific productivity of approximately 100 mg/g, as determined from the protein dry weight following purification.

The standard shake-flask production conditions used were: 50 mL of culture medium in 500 mL Erlenmeyer flasks at 37°C, 250 rpm (25 mm orbital) and with an initial culture medium pH of 7.0. Induction was carried out with 1 mM IPTG during the logarithmic or/and early stationary phases of growth with SELP-59-A production levels being tested (SDS-PAGE, see below) at various time points up to 16 hours after induction. The logarithmic and early stationary phases for each condition investigated were determined from pre-runs without any induction. Biomass production and pH were measured throughout the duration of the studies. In all cases, production cultures were inoculated to a starting biomass dry cell weight (DCW) of 0.05 g/L using the resuspended cell pellet of an overnight 25°C lysogeny-broth (LB: 10 g/L bacto tryptone; 5 g/L yeast extract; 5 g/L NaCl, pH 7.0) pre-culture.

#### Medium optimisation

A number of production media, as commonly described in literature for recombinant protein production in *E*. *coli*, were investigated as indicated above. The media tested and their preparation are described in Table 1. All media were used at pH 7.0 and sterilised by autoclaving at 121°C for 20 minutes, components such as phosphates and magnesium were filter sterilized and added separately after sterilisation. All media were supplemented with filter sterilised ampicillin at a final concentration of 200 μg/mL. Having selected the best producing medium we then attempted to optimise the composition of this medium. Varied concentrations of bacto-tryptone (from 5 to 20 g/L), yeast extract (from 15 to 50 g/L) and glycerol (0 – 10 g/L) were investigated. Varied concentrations of different carbon sources such as glucose (5 – 20 g/L) and fructose (5 – 20 g/L) were also examined. Finally, the effects of a range of additives such as NaCl (0 – 20 g/L), MgSO_4_ (0 – 15 mM), FeSO_4_ (0 – 0.5 mM), CaCl_2_ (0–0.05 g/L), (NH_4_)_2_SO_4_ (2 – 7 g/L), NH_4_OH (1 – 5 g/L) and the amino acids (2.5 – 10 mM) methionine, isoleucine, valine, glycine, alanine, proline and serine on SELP production were examined. All additives were filter sterilized before addition to the sterilised medium.

**Table 1:** Details of the media investigated

**Medium**	**Preparation**
Lysogeny broth (LB)	10 g/L bacto tryptone, 5 g/L yeast extract, 5 g/L NaCl
Lysogeny broth-Miller (LBM)	10 g/L bacto tryptone, 5 g/L yeast extract, 10 g/L NaCl, 0.98 g/L MgSO4
Terrific broth (TB)	5 g/L glycerol, 12 g/L bacto tryptone, 24 g/L yeast extract, 2.31 g/L KH_2_PO_4_ and 12.54 g/L K_2_HPO_4_
Terrific broth with lactose (TBlac)	5 g/L glycerol, 12 g/L bacto tryptone, 24 g/L yeast extract, 2.31 g/L KH_2_PO_4_ and 12.54 g/L K_2_HPO_4_, 2 g/L lactose
Modified terrific broth (TBmod)	0.5 g/L glucose, 12 g/L bacto tryptone, 24 g/L yeast extract, 6.8 g/L KH_2_PO_4_, 7.1 g/L Na_2_HPO4.12H_2_O, 0.15 g/L MgSO_4_, 3.3 g/L (NH_4_)_2_SO_4_
Terrific broth auto-induction medium (TBaim)	0.5 g/L glucose, 2 g/L lactose, 12 g/L bacto tryptone, 24 g/L yeast extract, 6.8 g/L KH_2_PO_4_, 7.1 g/L Na_2_HPO_4._12H_2_O, 0.15 g/L MgSO_4_ 3.3 g/L (NH_4_)_2_SO_4_
Super broth (SB)	5 g/l glycerol, 35 g/L bacto tryptone, 20 g/L yeast extract, 2.31 g/L KH_2_PO_4_ and 12.54 g/L K_2_HPO_4_
Modified super broth (SBmod)	0.5 g/L glucose, 35 g/L bacto tryptone, 20 g/L yeast extract, 6.8 g/L KH_2_PO_4_, 7.1 g/L Na_2_HPO_4._12H_2_0, 0.15 g/L MgSO_4_, 3.3 g/L (NH_4_)_2_SO_4_
Super broth auto-induction medium (SBaim)	0.5 g/L glucose, 2 g/L lactose, 35 g/L bacto tryptone, 20 g/L yeast extract, 6.8 g/L KH_2_PO_4_, 7.1 g/L Na_2_HPO_4_.12H_2_O, 3.3 g/L (NH_4_)_2_SO_4_; 0.15 g/L MgSO_4_
Super broth enriched (SBenrich)	20g/l fructose, 32 g/L bacto tryptone, 60 g/L yeast extract, 5 g/L NaCl
Modified super optimal broth (SOC)	4 g/L glucose, 20 g/L tryptone peptone, 5 g/L yeast extract, 0.58 g/l NaCl, 0.186 g/L KCl, 2.46 g/L MgSO_4_, 2 g/L MgCl_2_
New Brunswick Scientific medium (NBS)	25 g/L glucose, 5 g/L yeast extract, 2 g/L KH_2_PO_4_, 3 g/L K_2_HPO_4_, 0.5 g/L MgSO_4_.7H_2_O, 5 g/L (NH_4_)_2_HPO_4_, 1 mg/L thiamine, 3 mL/L Holmes trace elements solution [46]
Modified New Brunswick Scientific medium (NBSmod)	5 g/L glycerol, 5 g/L yeast extract, 2 g/L KH_2_PO_4_, 3 g/L K_2_HPO_4_, 0.5 g/L MgSO_4_.7H_2_O, 5 g/L (NH_4_)_2_HPO_4_, 1 mg/L thiamine, 3 mL/L Holmes trace elements solution [46]
ZYB (ZYB)	10 g/L NZ-amine, 5 g/L yeast extract, 5 g/L NaCl
ZYB buffered (ZYBbuff)	10 g/L NZ-amine, 5 g/L yeast extract, 2.31 g/L KH_2_PO_4_, 12.54 g/L K_2_HPO_4,_ 5 g/L NaCl
Minimal medium M9 (M9)	10 g/L glucose, 0.85 g/L Na_2_HPO_4_.12H_2_O; 1 g/L NH_4_Cl; 3 g/L KH_2_PO_4_; 0.5 g/L NaCl; 0.24 g/L MgSO4
Riesenberg minimal medium (Ries)	20 g/L glucose, 13.3 g/L KH_2_PO_4_, 4 g/L (NH_4_)_3_PO_4_, 1.7 g/L citric acid, 0.24 g/L MgSO4, 3 mL Holmes trace element solution [46]

All media were adjusted to pH 7.0 and sterilised by autoclaving at 121°C for 20 minutes, components such as phosphates and magnesium were filter sterilized and added separately after sterilisation. All media were supplemented with filter sterilised ampicillin at a final concentration of 200 μg/mL following sterilisation.

#### Temperature

The effect of temperature on growth and production was investigated as indicated above with cultures being incubated at 25, 30, 37 or 42°C for the duration of the production. Shifts in temperature, at the time of induction, from 25 to 30°C, 25 to 37°C, 30 to 37°C, 37 to 42°C and 37 to 30°C were also investigated.

#### pH

Initial culture pHs of 5.5 to 9.5 at 0.5 pH unit intervals were tested.

#### Shake flask volume:medium volume

The effects of varying the flask volume to medium volume ratio from 3:1 (167 mL medium in 500 mL Erlenmeyer) to 5:1 (100 mL medium), 10:1 (50 mL medium) and 20:1 (25 mL medium) were examined.

#### Agitation

Shake flask agitation rates of 150 rpm, 200 rpm and 250 rpm on a 25 mm orbital Novotron AK82 incubator-shaker (Infors HT) were investigated.

#### Elapsed fermentation time at induction and induction period

For optimisation of the EFT at induction and the induction period, shake flask cultures were induced with 1 mM IPTG during the exponential (2 hrs. EFT, 0.75 g/L), declining exponential (4 and 6 hrs. EFT, 2 and 3 g/L), early stationary (8 hrs. EFT, 4 g/L), stationary (10 hrs. EFT, 4 g/L) and the late stationary (14 hrs. EFT, 5 g/L) phases of growth. SELP production levels were compared for samples taken at induction periods of 2, 4, 6, 9 and 16 hours after induction.

#### IPTG induction concentration

Final IPTG concentrations of 0.1, 0.5, 1 and 3 mM were investigated.

### Analysis of SELP production: comparative SDS-PAGE

0.5 mL culture samples were taken at various time points throughout the shake flask productions for a comparative analysis of total SELP production levels. Cell pellets were collected by centrifugation for 5 minutes at 14000 × g and after overnight storage at −20°C were resuspended in 500 μL TE buffer (50 mM Tris–HCl, 1 mM EDTA, pH 8.0) and 125 μL SDS-PAGE loading solution (100 g/L sodium dodecyl sulphate, 10 mM β-mercaptoethanol, 200 g/L glycerol, 0.2 M Tris–HCl, 0.5 g/L bromophenol blue, pH 6.8). After vigorous vortexing, suspensions were centrifuged at 14000 × g for 25 minutes to remove cellular debris and the supernatant loaded on 10% SDS-PAGE gels. Standard protocols, essentially as described by Laemmli [47] were used in the preparation and running of the SDS-PAGE. Equal volumes (typically 4 μL) of each sample were loaded on the gels and protein bands were visualised by negative staining with 0.3 M copper chloride solution, with immediate mixing. A comparative evaluation of the production levels was carried out by densitometry analysis using the ImageJ 1.45s software (NIH, USA).

The best EFT for induction and induction period for each parameter variant was first determined and these ‘best producers’ were then compared, where possible, on the same SDS-PAGE gel. In those cases where more parameter variants than possible to load on a single gel were encountered (i.e. media optimisation), each gel was also loaded with an internal standard of approximately 3 μg of purified SELP to allow for comparisons between gels.

Comparison of the SELP-59-A band intensity for each of the parameter variants investigated to that for the optimised standard condition (500 mg/L volumetric productivity, 100 mg/g specific productivity) allowed for calculation of the volumetric and specific productivities. Specific productivity was calculated at the point of maximum volumetric productivity.

### Analytical methods

Biomass dry cell weights (DCW, g/L) were determined from the weights of washed pellets of 2 mL culture samples dried overnight at 70°C.

Organic acid (acetate) and carbohydrate (glycerol, glucose, fructose) levels in culture supernatants were monitored using a Rezex™ 8 μm ROA-organic acid H+(8%) high performance liquid chromatography column (Phenomenex). 2.5 mM H_2_SO_4_ was used for the mobile phase, the column was maintained at 60°C and detection was by refractive index measurement with an Elite LaChrom L-2490 RI detector (VWR Hitachi) at 40°C. An Elite LaChrom (VWR Hitachi) chromatography system was used with the EZChrom Elite 3.3.2 SP2 software for date collection and analysis.

Total phosphate concentration in culture supernatant was measured by the phosphate assay described by Chen et al. [48].

Ammonia-nitrogen concentration was determined by the Berthelot colour reaction as previously described [49].

Percentage plasmid stability in culturable cells was determined by expressing the number of colonies enumerated on LB agar plates containing ampicillin (LB agar: 10 g/L bacto tryptone; 5 g/L yeast extract; 5 g/L NaCl, 20 g/L agar, 100 μg/mL ampicillin) as a percentage of the number of colonies enumerated on LB agar plates without ampicillin. Culture samples were taken at various time points throughout the shake flask productions with 50 μL of serial dilutions being plated in triplicate on both types of plate cultures before incubation overnight at 37°C.

A disk diffusion assay was developed for monitoring ampicillin concentration during the fermentations. The β-lactamase present in the culture supernatants was immediately removed after sample collection by filtering through a 5 KDa MWCO Vivaspin 4 column (GE Healthcare). Antimicrobial susceptibility test discs (Oxoid) were then soaked in 21 μL of the filtrate and placed on LB agar plates overlayed with a 0.8% agar layer containing the *E*. *coli* BL21(DE3) test organism at a concentration of 1×10^7^ cfu/mL. Cultures were incubated at 37°C overnight and inhibition halos measured and compared to controls containing 0–200 μg/mL ampicillin. The sensitivity of the assay was increased below 40 μg/mL by concentrating the filtrates by freeze drying prior to loading the discs.

### Effects of acetic acid on *E*. *coli* BL21 (DE3) growth

The effect of acetic acid on the expression host under the conditions used was examined by adding 0.5 to 7 g/L of filter sterilised acetic acid to the production cultures at 0, 2 or 4 hours incubation. The culture growth (DCW) and pH were monitored every 30 minutes and compared to that of cultivations without added acetic acid. Acetic acid concentrations were verified by HPLC as indicated above.

### Purification of SELP

SELP was purified as previously described [16]. Briefly, the intracellularly produced protein was liberated by sonication and purified by a combination of overnight treatment at pH 3.5 followed by 20% ammonium sulphate treatment of the supernatant. The ammonium sulphate precipitate was then extensively dialysed in water before lyophilisation. Protein yield was determined from the weight of the dried purified product.

## Abbreviations

SELP: Silk-elastin-like protein; SELP-59-A: Silk-elastin-like protein of the present study (see Figure 1 for details); rpm: Rotations per minute; IPTG: Isopropyl β-D-1-thiogalactopyranoside; PBP: Protein based polymers; GAGAGS: Silkworm silk consensus sequence block composed of the amino acids: Glycine Alanine, Glycine, Alanine, Glycine and Serine.; VPGVG: Mammalian elastin repetitive sequence block composed of the amino acids: Valine Proline, Glycine, Valine, and Glycine; LB: Lysogeny broth; LBM: Lysogeny broth-Miller; TB: Terrific broth; SB: Super broth; TBlac: Terrific broth with lactose; TBmod,: Modified terrific broth; TBaim: Terrific broth auto-induction medium; SBmod: Modified super broth; SBaim: Super broth auto-induction medium; SB enrich: Super broth enriched; SOC: Modified super optimal broth; NBS: New Brunswick Scientific medium; NBSmod: Modified New Brunswick Scientific medium; ZYB: ZYB medium; ZYBbuff: ZYB buffered; M9: Minimal medium M9; Ries: Riesenberg minimal medium; DCW: Biomass Dry Cell Weight (g/L); EFT: Elapsed fermentation time (hours); SDS-PAGE: Sodium dodecyl sulfate polyacrylamide gel electrophoresis; OFAT: One factor at a time experimental approach; MWM: Broad range protein molecular weight marker (ThermoScientific).

## Competing interests

The authors declare that they do not have any competing interests.

## Authors’ contributions

TC carried out the empirical studies, the HPLC analysis, the investigation of the effects of acetic acid and drafted the manuscript. AC and JS carried out the empirical studies, the HPLC analysis, the characterisation of the optimised process and all protein purifications. FB participated in the empirical studies, the characterisation of the optimised process and the antibiotic concentration determination studies. RM carried out the investigation of the effects of agitation and provided guidelines for protein purification. MC conceived the study and participated in its design and implementation. All authors read and approved the final manuscript.
